# Programmed gRNA Removal System for CRISPR-Cas9-Mediated Multi-Round Genome Editing in *Bacillus subtilis*

**DOI:** 10.3389/fmicb.2019.01140

**Published:** 2019-05-21

**Authors:** Hayeon Lim, Soo-Keun Choi

**Affiliations:** ^1^Infectious Disease Research Center, Korea Research Institute of Bioscience and Biotechnology, Daejeon, South Korea; ^2^Department of Biosystems and Bioengineering, KRIBB School of Biotechnology, Korea University of Science and Technology, Daejeon, South Korea

**Keywords:** *Bacillus subtilis*, CRISPR/Cas9, self-curing, genome editing, extracellular protease

## Abstract

CRISPR/Cas9 has become a simple and powerful genome editing tool for many organisms. However, multi-round genome editing should replace single-guide RNA (sgRNA) every round, which is laborious and time-consuming. Here, we have developed a multi-round genome editing system in which genome editing and the programmed removal of the sgRNA have sequentially occurred in a growth-dependent manner in *Bacillus subtilis*. The system contains two plasmids, one containing a *cas9* gene and the other containing two sgRNAs and a donor DNA for homology directed repair (HDR). The two sgRNAs are chromosome-targeting (sgRNA_ct_) and self-targeting (sgRNA_st_) under the control of a constitutive promoter and sporulation-specific promoter, respectively. In the growth phase, the sgRNA_ct_ is transcribed and complexed with the Cas9 to edit the chromosomal target, while the sgRNA_st_ is transcribed in the sporulation phase and complexed with the Cas9 to attack its own plasmid. Therefore, the system automatically makes the cell ready for next-round genome editing during cultivation. The system was approved through the sequential deletion of eight extracellular protease genes in the *B. subtilis*, suggesting that it can be used for versatile applications in multi-round genome editing.

## Introduction

A type II clustered regularly interspaced short palindromic repeat (CRISPR)/Cas9 system of *Streptococcus pyogenes*, derived from the bacterial adaptive immune system has been developed into convenient genome engineering tools for diverse organisms, such as *Escherichia coli* (Jiang et al., 2015), *Streptomyces* spp. (Cobb et al., 2014), *Saccharomyces cerevisiae* (DiCarlo et al., 2013), mice (Shen et al., 2013), *Bomyx mori* (Wang et al., 2013), Drosophila (Bassett et al., 2013), crop plants (Shan et al., 2013), and human cell lines (Mali et al., 2013). The CRISPR/Cas9 system requires the CRISPR-associated protein (Cas9), a trans-activating CRISPR RNA (tracrRNA), and a CRISPR RNA (crRNA). A synthetic single guide RNA (sgRNA) is constructed by fusing together the tracrRNA and the crRNA. The endonuclease Cas9 and sgRNA, including a 20 bp target sequence, which is decided by protospacer adjacent motif (PAM), are enough to make a double-strand DNA break (DSB) in a specific region of the genome (Jinek et al., 2012; Barrangou and Marraffini, 2014). The DSB can be repaired using two mechanisms—the homology directed repair (HDR) and the non-homologous end joining (NHEJ). For the HDR pathway, donor DNA fragment that is homologous to the region flanking DSB site is required to repair the cleavage site. The NHEJ system repairs the broken end without the donor DNA and results in the insertion or deletion (indel) mutations. The CRISPR/Cas9 systems can provide an efficient tool for editing the genome (Doudna and Charpentier, 2014).

Several types of CRISPR/Cas9-based genome editing system have been reported in *B. subtilis*, including chromosome-integrated method, single-, or two-plasmid systems (Hong et al., 2018). The chromosome-integrated CRISPR/Cas9 was used for single or double gene mutation, and for the insertion of a 2.9 kb hyaluronic acid biosynthetic operon. However, this method has a limitation – the gRNA integration site must be restored to its original form in order to introduce a new gRNA cassette (Westbrook et al., 2016). A single-plasmid-based method was used for the disruption of multiple genes, which are *srfC*, *spoIIAC*, *nprE*, *aprE*, and *amyE*, with an efficiency of 33–53% (Zhang et al., 2016). In addition, a two-plasmid CRISPR/Cas9 system was introduced for *spo0A* deletion, *sigE* point mutation, *gfp* gene insertion, and large-sized gene deletion in *B. subtilis* (So et al., 2017). The two-plasmid system showed the highest mutation efficiency. However, the plasmid-based gene editing systems still require an iterative process of removing the plasmid containing the previous sgRNA, and for introducing the plasmid carrying a new sgRNA for the multi-round genome editing. The previous methods for plasmid removal were usually dependent on a temperature sensitive replication origin or a traditional negative selection method, which are labor-intensive and time-consuming (Trevors, 1986). Therefore, a method that can remove the specific plasmid DNA with high efficiency would facilitate multi-round genome editing.

In *E. coli*, CRISPR/Cas9-mediated plasmid curing methods have used replicon, or antibiotic resistant marker-targeting sgRNAs for plasmid recycling (Jiang et al., 2015; Li et al., 2015; Ronda et al., 2016; Lauritsen et al., 2017). These systems have used inducible promoters for the controlled transcription of the sgRNAs in order to avoid the transcription of the self-targeting sgRNA (sgRNA_st_), prior to chromosome-targeting sgRNA (sgRNA_ct_). However, the CRISPR/Cas9-mediated plasmid curing has not yet been reported in *B. subtilis*. Several inducible promoters have been used in the *B. subtilis*, such as P_spac_ and P_xyl_. However, they have substantial basal expression levels despite the absence of an inducer (Bhavsar et al., 2001). Using a sporulation-specific promoter for tightly controlled transcription of the sgRNA_st_, we have developed here a CRISPR/Cas9-based multi-round genome editing system in which the genome editing and automatic plasmid curing occur in sequence during cultivation. The system was used to construct a *B. subtilis* mutant, containing eight extracellular proteases’ deletion without the remainder of any foreign DNA trace.

## Materials and Methods

### Strains and Culture Conditions

The plasmids and *B. subtilis* strains used in this study are listed in Table 1. *E. coli* MC1061 was used to construct the recombinant plasmids. *B. subtilis* cells were cultured in Luria-Bertani (LB), LB agar (Difco Laboratories, Detroit, MI, United States) and 2×GYS (2 g/L glucose, 4 g/L (NH_4_)_2_SO_4_, 4 g/L yeast extract, 1 g/L K_2_HPO_4_, 0.82 g/L MgSO_4_⋅7H_2_O, 0.16 g/L CaCl_2_⋅2H_2_O, and 0.14 g/L MnSO_4_⋅5H_2_O, pH 7.0) for sporulation at 37°C. To test the efficiency of the plasmid cleavage, 100 mM IPTG (Isopropyl β-D-1-thiogalactopyranoside) was added to the 2×GYS (final concentration of 1 mM), named 2×GYS-I. When required, the antibiotics were supplemented with ampicillin (100 μg/ml), neomycin (10 μg /ml) or chloramphenicol (5 μg /ml). Transformation of *B. subtilis* was carried out utilizing the two-step transformation procedure (Harwood and Cutting, 1990) except that the EGTA (ethylene glycol-bis(2-aminoethyl ether) N, N, N′, N′-tetraacetic acid) was not used.

**Table 1:** *Bacillus* strains and plasmids used in this study.

Strain/plasmid	Genotype/description	References
***BACILLUS* STRAINS**		
*B. subtilis* 168	Tryptophan auxotrophic (trpC2)	Laboratory stock
BS-C100	*B. subtilis* 168 containing plasmid pHCas9	So et al., 2017
BS-D119a	BS-C100 containing plasmid pSC1	This study
BS-D119b	BS-C100 Δ*aprE* containing plasmid pG2-aE	This study
BS-D119c	BS-C100 Δ*aprE* containing plasmid pG-aE	This study
BS-D119	BS-C100 Δ*aprE*	This study
BS-D120	BS-C100 Δ*aprE* Δ*nprE*	This study
BS-D121	BS-C100 Δ*aprE* Δ*nprE* Δ*epr*	This study
BS-D122	BS-C100 Δ*aprE* Δ*nprE* Δ*epr* Δ*bpr*	This study
BS-D123	BS-C100 Δ*aprE* Δ*nprE* Δ*epr* Δ*bpr* Δ*mpr*	This study
BS-D124	BS-C100 Δ*aprE* Δ*nprE* Δ*epr* Δ*bpr* Δ*mpr* Δ*nprB*	This study
BS-D125	BS-C100 Δ*aprE* Δ*nprE* Δ*epr*Δ*bpr* Δ*mpr* Δ*nprB* Δ*vpr*	This study
BS-D126	BS-C100 Δ*aprE*Δ*nprE*Δ*epr* Δ*bpr* Δ*mpr* Δ*nprB* Δ*vpr* Δ*wprA*	This study
**PLASMIDS**		
pAD123	*E. coli*-*Bacillus* shuttle vector	Bacillus Genetic Stock Center
pAgR	Plasmid pAD123 derivative containing synthetic sgRNA module	So et al., 2017
pSC1	Plasmid pAD123 derivative containing sgRNA module with 20 bp self-targeting gRNA under the control of sporulation-specific promoter P_spoIV A_	This study
pG2	Plasmid pSC1 containing synthetic sgRNA module under the control of the constitute promoter (Para)	This study
pG2-aE	Plasmid pG2 containing *aprE*-targeting 20 bp gRNA and 1 kb Donor DNA	This study
pG-aE	Plasmid pG2-aE without sporulation-specific promoter P_spoIV A_ and 20 bp self-targeting gRNA	This study
pG2-nE	Plasmid pG2 containing *nprE*-targeting 20 bp gRNA and 1 kb Donor DNA	This study
pG2-er	Plasmid pG2 containing *epr*-targeting 20 bp gRNA and 1 kb Donor DNA	This study
pG2-br	Plasmid pG2 containing *bpr*-targeting 20 bp gRNA and 1 kb Donor DNA	This study
pG2-mr	Plasmid pG2 containing *mpr*-targeting 20 bp gRNA and 1 kb Donor DNA	This study
pG2-nB	Plasmid pG2 containing *nprB*-targeting 20 bp gRNA and 1 kb Donor DNA	This study
pG2-vr	Plasmid pG2 containing *vpr*-targeting 20 bp gRNA and 1 kb Donor DNA	This study
pG2-wA	Plasmid pG2 containing *wprA*-targeting 20 bp gRNA and 1 kb Donor DNA	This study

### Plasmids Construction

The primers used in this study are listed in Table 2. The sporulation-specific promoter (P_spoIV A_) and sgRNA_st_, containing 20 bp of self-targeting sequences, was obtained through a fusion polymerase chain reaction (PCR) with primers spoIVA-rep-F1 and spoIVA-rep-R1. The PCR product was digested with EcoRI and BamHI and inserted into corresponding sites of plasmid pAD123 (Jeong D.E. et al., 2018) to construct the pSC1. To introduce the synthetic sgRNA module under the control of the constitute promoter (P_ara_) into the plasmid pSC1, primers g-AarI-F1 and g-AarI-R1 were used to amplify the promoter and the synthetic sgRNA module, using the pAgR (So et al., 2017) as a template. The PCR product was digested with BglII and NsiI and ligated with large a fragment of BamHI- and NsiI-digested pSC1, to construct plasmid pG2.

**Table 2:** Oligonucleotides and Primers used in this study.

Oligonucleotide	Sequence (5′→3′)
aprE-gF1	Attg**accgattgagttattaagag**
aprE-gR1	Aaac**ctcttaataactcaatcggt**
nprE-gF1	Attg**agacaagcgtgcccggaagg**
nprE-gR1	Aaac**ccttccgggcacgcttgtct**
epr-gF1	Attg**ttaaaccagtattatgcaac**
epr-gR1	Aaac**gttgcataatactggtttaa**
bpr-gF1	Attg**ataacggaaagacatcaagc**
bpr-gR1	Aaac**gcttgatgtctttccgttat**
mpr-gF1	Attg**tatccgtacggtacttattc**
mpr-gR1	Aaac**gaataagtaccgtacggata**
nprB-gF1	Attg**tctcaactgatcgggtatac**
nprB-gR1	Aaac**gtatacccgatcagttgaga**
vpr-gF1	Attg**aaagtcgccgttgtcaaacg**
vpr-gR1	Aaac**cgtttgacaacggcgacttt**
wprA-gF1	Attg**atattcagtacccttatcaa**
wprA-gR1	Aaac**ttgataagggtactgaatat**

**Primer**	**Sequence (5′→3′)**

spoIVA-rep-F1	Caagaattcgatgtcatattcaaataggacaacgtcatacacatatagtgca aatttctgcgatgatggagttttagagctagaaatagcaagttaaa
spoIVA-rep-R1	Agaggatccaaaaaaagcaccgactcggtgccactttttcaagttgataac ggactagccttattttaacttgctatttctagctctaaaactccat
g-AarI-F1	Taagatctaagcttaaagattgacagtataatagtc
g-AarI-R1	Aaaatgcatactagtagctagcaggatccaaaaaaagcaccgactc
aprE-NF1	Tggatcctagttatttcgagtctctacgg
aprE-NR1	tgtgcaatatgatcttcttcc
aprE-CF1	Ggaagaagatcatattgcacatcggctaccctgcaaaatat
aprE-CR1	Atactagtcggtgcttgtgaagattttca
nprE-NF1	Tggatccttatcaatcagcctgccaggt
nprE-NR1	Taagcaagacgatagctgcc
nprE-CF1	Ggcagctatcgtcttgcttatgatggcgacggttcattctt
nprE-CR1	Atactagtcttcaactttagcggcatca
epr-NF1	Gcagaccaggagacagtaaaa
epr-NR1	Tcgtgccaaggctcatattga
epr-CF1	Tcaatatgagccttggcacgatgccgaactccgacgccaaaa
epr-CR1	Atactagttcttcactttgtctaaccgtt
bpr-NF1	Tggatccatttcctgattcaccgaataa
bpr-NR1	Cggaactccccattcccagtt
bpr-CF1	Aactgggaatggggagttccgatcagaacaaggctatacag
bpr-CR1	Atactagtataaccgacgaaaggcttcaa
mpr-NF1	Tggatcctgctgctgattcagttgaaa
mpr-NR1	Ggctgtaaggtttgaatgttt
mpr-CF1	Aaacattcaaaccttacagccctgaaacgtataagctgacct
mpr-CR1	Atactagtgagatgtgatgggttactgat
nprB-NF1	Tgctagcatcaaaccccttcatacatca
nprB-NR1	Cagatgtcgagagtctcacaa
nprB-CF1	Ttgtgagactctcgacatctgatgaaatcacacacgcagtca
nprB-CR1	Atactagtatagaatgccgacagcctca
vpr-NF1	Tggatcctctccgcaaatggatgacagt
vpr-NR1	Ctgttgccgtttgaggtaac
vpr-CF1	Gttacctcaaacggcaacagtgttatggatacgtggatgat
vpr-CR1	Atactagttacttttgcagtggctttccc
wprA-NF1	Tggatccggttgaaatgagtgtcgatca
wprA-NR1	Ttatgtacggatgagaggct
wprA-CF1	Agcctctcatccgtacataattgcagcccaaagcgataac
wprA-CR1	Atactagtagcttaggattttgagcaaac

*Underlined sequences are the restriction enzyme sites. Bolded sequences represent the 20 bp synthetic gRNA.*

The 20 bp gRNA-containing oligonucleotides were generated by mixing synthetic primers x-gF1 and x-gR1 (× indicates the target genes; *aprE, nprE, epr, bpr, mpr, nprB, vpr*, and *wprA*). The oligonucleotides were ligated with AarI-digested pG2 to produce eight pG2 derivatives. For further cloning of donor DNAs, 500 bp fragments of each of the N- and C-terminus of the target site were amplified using the *B. subtilis* 168 chromosome as a template, with primer sets x-NF1/x-NR1 and x-CF1/x-CR1. The fusion PCR of the two DNA fragments, with primers x-NF1/x-CR1, resulted in a 1 kb donor DNA. Then the donor DNAs were digested with BamHI and SpeI and ligated with the corresponding the pG2 derivatives to convert pG2-aE to pG2-wA (NheI instead of BamHI in pG2-nB) (Table 1). To construct pG-aE, EcoRI- and HindIII-, digested pG2-aE was treated with dNTPs and the Klenow fragment, followed by blunt end ligation.

### Serial Gene Editing in *B. subtilis*

BS-C100, a *B. subtilis* 168 derivative carrying pHCas9 (So et al., 2017) was transformed with the sgRNAs-containing plasmids. The transformed cells were spread on LB agar plate supplemented with chloramphenicol (5 μg/ml) and neomycin (10 μg/ml). One colony selected from the plate was cultured in the 2×GYS -I medium containing neomycin (10 μg/ml) for 16 h. Subsequently, the cells were heat-treated at 80°C for 1 h and spread on the LB agar plate containing neomycin (10 μg/ml). The colonies on the plate were observed for antibiotic sensitivity to chloramphenicol (5 μg/ml) to select the colonies that have been removed from the sgRNAs-containing plasmid. The mutations were confirmed by the colony of PCR, DNA sequencing, and protease assay.

### Protease Assay

Protease assay was performed using FTC (fluorescein isothiocyante)-casein as a substrate provided by the Pierce Fluorescent Protease Assay Kit (Thermo Scientific, Rockford, IL, United States). *Bacillus* cells were cultured in LB medium for 16 h at 37°C. Following this, the culture supernatants were collected by centrifugation at 10,000 × *g* for 5 min. The FTC-casein working reagent (100 μl) was mixed with 100 μl of the diluted supernatants (2^−8^) and incubated at room temperature for 24 h. The 10% trichloroacetic acid (400μl) was added to the mixture and incubated at ambient temperature for 5 min. After centrifugation at 10,000 × *g* for 5 min, 300 μl of the supernatant was mixed with 900 μl of 0.5 M Tris, pH 9, to measure fluorescence. Fluorescence intensity (excitation, 485 nm; emission, 535 nm) was measured using the TriStar^2^ LB 942 Multimode Reader (Berthold Technologies GmbH & Co. KG, Bad Wildbad, Germany). Blank fluorescence was subtracted from each measurement and the fluorescence reading was normalized to OD_600_ = 1.

## Results

### Construction of the Self-Targeting sgRNA Module

Most of the CRISPR/Cas9-based genome editing in *B. subtilis* has been performed through plasmid-mediated methods (Altenbuchner, 2016; Zhang et al., 2016; Burby and Simmons, 2017; So et al., 2017). In multi-round genome editing, the elimination of sgRNA-containing plasmid is essential for the following round. It was reported that the CRISPR/Cas9 system using sgRNA_st_ was used as a tool for plasmid removal in *E. coli* (Jiang et al., 2015). However, the transcription of the sgRNA_st_ needs to be tightly regulated because the unnecessary removal of sgRNA_st_-containing plasmid through leaky transcriptions may result in the simultaneous removal of sgRNA_ct_, which can hinder the genome editing.

**FIGURE 1:**
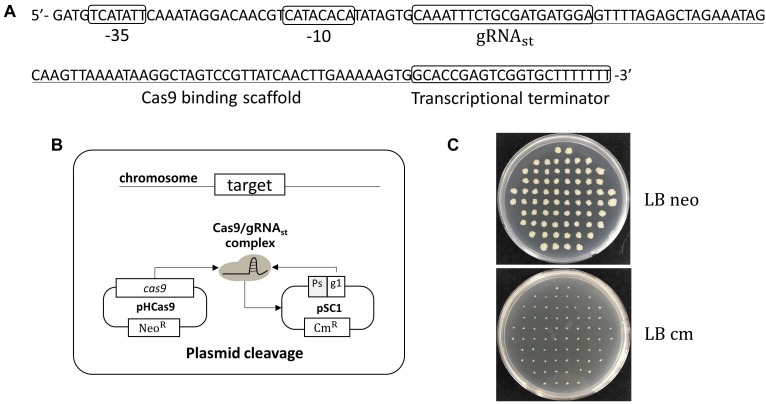
Development of plasmid curing system using Cas9 and self-targeting sgRNA (sgRNA_st_). **(A)** Sequence of a self-targeting sgRNA under the control of P_spoIV A_ promoter, 20 bp self-targeting gRNA (gRNA_st_), Cas9 binding scaffold, and transcriptional terminator. **(B)** Scheme of the plasmid self-curing. Introduction of plasmid pSC1 into strain BS-C100 and incubation in sporulation medium lead to the removal of pSC1; Ps and g1 indicate sporulation-specific promoter and gRNA_st_, respectively. **(C)** After self-curing, all *B. subtilis* colonies were grown on LB neomycin (LB neo) plates, but did not grow on LB chloramphenicol (LB cm) plates.

In *B. subtilis*, the expression of sporulation-relating genes are tightly regulated by the phosphorylation of the master regulator Spo0A, and by the cascade activation of sporulation-specific sigma factors, σ^*F*^, σ^*E*^, σ^*G*^, and σ^*K*^ (Piggot and Hilbert, 2004). Thus, a sporulation-specific promoter (P_spoIV A_) (Roels et al., 1992; Hilbert and Piggot, 2004) was selected for the tight-control transcription of the sgRNA_st_. The sgRNA_st_ contains 20 bp gRNA sequence targeting replication origin of plasmid pAD123 (Figure 1A). To confirm the self-curing system, the *B. subtilis* strain BS-C100 containing pHCas9 (So et al., 2017) was transformed with the plasmid pSC1 containing sgRNA_st_. The resulting strains of BS-D119a were cultured for 16 h in a sporulation medium (2×GYS) containing neomycin. Following the heat treatment, the cells were spread on the LB neomycin or chloramphenicol agar plate. We expected that the sgRNA_st_/Cas9 complex would attack its own replication origin during the sporulation phase to exhibit chloramphenicol sensitivity (Figure 1B). All seventy colonies selected for antibiotic susceptibility tests showed sensitivity to chloramphenicol, indicating that the curing system works well with 100% efficiency (Figure 1C).

### Growth-Phase Dependent Automatic sgRNAs-Exclusion System

**FIGURE 2:**
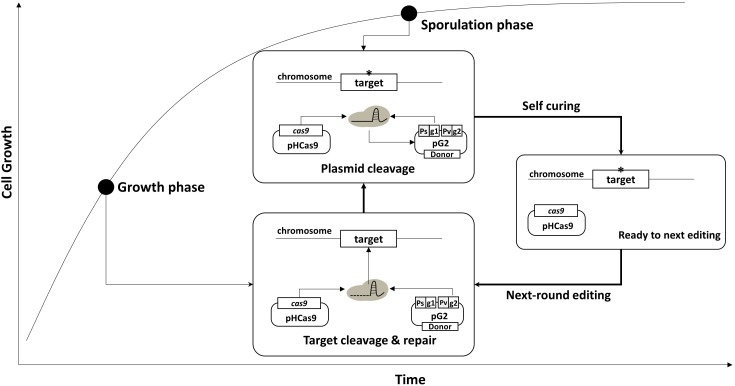
Strategy for serial genome editing in *B. subtilis*. The pG2 plasmid containing sgRNA_ct_, sgRNA_st_, and donor DNA fragment is introduced into pHCas9-containing cells. In the growth phase, sgRNA_ct_/Cas9 complex is responsible for target gene editing. In the sporulation phase, sgRNA_st_/Cas9 complex make double strand breaks in the replication origin (rep) of the pG2 for self-curing. Thus, the cells are ready for the next round of editing which can be transformed with the new pG2 plasmid.

Using the curing system, we developed an efficient, multi-round genome editing process for *B. subtilis* (Figure 2). The system contains two plasmids: One carries a *cas9* gene, and the other contains a donor DNA and two sgRNAs – sgRNA_st_ and sgRNA_ct_. In the growth phase, Cas9 and sgRNA_ct_ are synthesized by the constitutive promoter and complexed to cleave the chromosomal target. The target editing occurs through a homologous recombination between the target and the donor DNA. In the sporulation phase, the sgRNA_st_ is synthesized and complexed with the Cas9 to cleave the sgRNAs-bearing plasmid. The resulting sgRNA-free cells are ready for the next round of editing.

**FIGURE 3:**
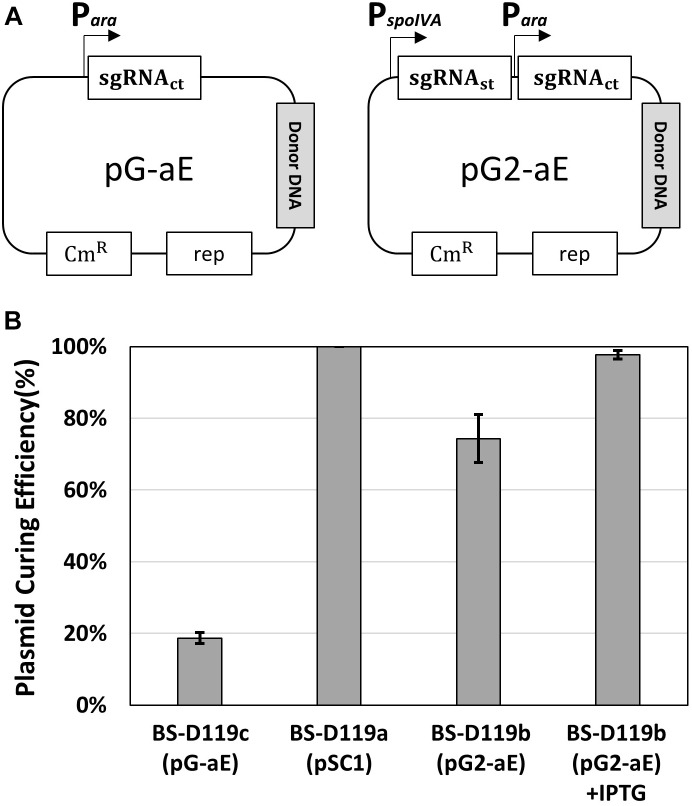
Plasmid curing efficiency, according to the existence of the sgRNA_st_. **(A)** The construction of two plasmids pG-aE and pG2-aE for *aprE* gene deletion and self-curing. **(B)** The comparison of plasmid curing efficiency. The use of IPTG could increase the curing efficiency of pG2-aE from 74 to 98%. The bars display the means of three independent experiments, with the error bars indicating standard deviations.

To confirm the system, two plasmids – pG-aE and pG2-aE – were constructed (Figure 3A). The pG-aE contains *aprE*-targeting sgRNA, under the control of constitutive a P_ara_ promoter (So et al., 2017) and the donor DNA, while the pG2-aE has the donor DNA and two sgRNAs—the *aprE*- and self-targeting sgRNAs. After introducing the two plasmids into the BS-C100, transformants were used to measure the efficiency of the *aprE* gene deletion and sgRNA-containing plasmid curing. Both plasmids showed similar *aprE* deletion efficiencies (80%), but different plasmid curing efficiencies. The curing efficiency of the plasmid pG-aE, which carries no sgRNA_st_, was less than 20%, while the efficiency of the plasmid pG2-aE, which contains both sgRNA_ct_ and sgRNA_st_, showed 74%. The curing efficiency of pG2-aE was reduced compared to the plasmid pSC1 containing only sgRNA_st_, which exhibited 100% efficiency. However, the efficiency was restored to 98% when the *cas9* expression was induced by the IPTG (Figure 3B). The results showed that our system can induce both genomic target-editing and removal of sgRNA-containing plasmids, in a growth-phase dependent manner, and the curing efficiency can be increased by the overexpression of the *cas9* gene.

### Consecutive Deletion of Eight Extracellular Protease Genes

**FIGURE 4:**
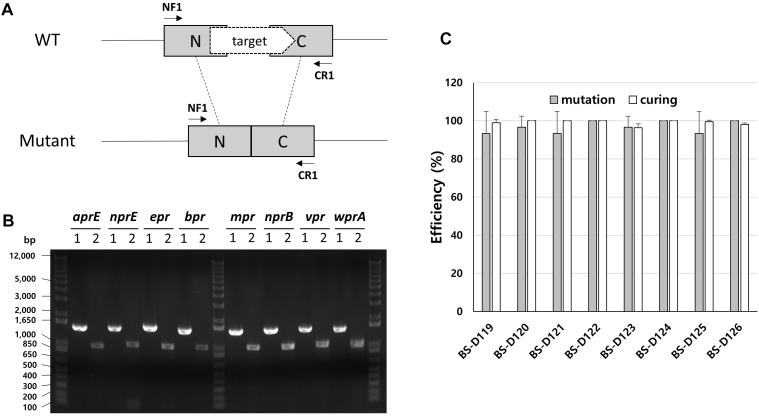
Deletion of eight extracellular protease genes in *B. subtilis*. **(A)** The chromosome structure of wild type strain (*B. subtilis* 168) and the deletion mutants of protease genes. The squared N and C indicate 500 bp donor DNA of each N- and C-terminus. The arrows indicate primer binding regions. **(B)** PCR analysis for confirming the deletion of each extracellular protease gene, using primer sets NF1/CR1. 1 indicates a wild type strain and 2 indicates strain BS-D126. Expected sizes of PCR products are 1.5 and 1.0 kb from wild type and mutant, respectively. **(C)** The mutation (gray) and curing (white) efficiencies of each mutant strain. The bars display the means of three independent experiments, with the error bars indicating standard deviations.

**FIGURE 5:**
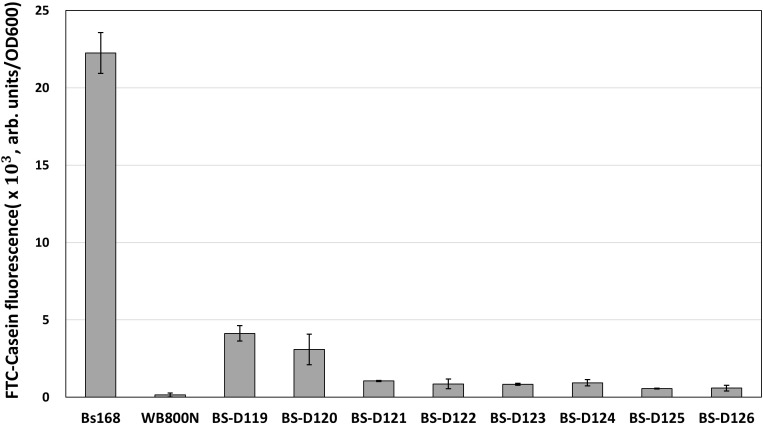
The protease assay using FTC-Casein as substrate for confirming the serial deletion of extracellular protease genes. The protease activity of deletion mutants is compared to the wild type strain (*B. subtilis* 168) and the WB800N. The protease activity was assessed as changes in fluorescence through the digestion of the FTC-Casein substrate. The bars display the means of three independent experiments, with the error bars indicating standard deviations.

*B. subtilis* has eight extracellular proteases known as: *aprE*, *nprE*, *epr*, *bpr*, *mpr*, *nprB*, *vpr*, and *wprA*. Extracellular protease deficient *B. subtilis* strains, such as WB800, were constructed to enhance the stability of the secreted heterologous proteins (Westers et al., 2006; Phuong et al., 2012). The WB800 strain carries antibiotic resistance markers that confer resistances to bleomycin, blasticidin S, hygromycin, and chloramphenicol (Wu et al., 2002). The chloramphenicol resistance gene was disrupted due to the insertion of neomycin resistance genes in the *B. subtilis* WB800N strain (Jeong H. et al., 2018). The construction of an eight, extracellular protease deficient mutant can be a good example for demonstrating our system. Eight plasmids (from pG2-aE to pG2-wA) were constructed to perform the consecutive deleting of the eight protease genes (Table 1). We sequentially introduced the eight plasmids into the BS-C100. Through the repetitive process of genome editing and plasmid self-curing, eight strains from BS-D119 to BS-D126 were obtained. Eight protease deficiencies of the final strain BS-D126 were confirmed by the PCR, using eight primer sets x-NF1/x-CR1 and DNA sequencing (Figure 4A,B). The efficiencies of gene deletion and plasmid curing of each mutant strain did not change significantly (Figure 4C). The protease assay showed that its extracellular activity was cumulatively decreased by the sequential removal of eight extracellular protease genes (Figure 5).

## Discussion

The genome editing of *B. subtilis* has been achieved by using antibiotic resistance markers for positive selection. However, *B subtilis* is a generally recognized as safe (GRAS) microorganism and has been used on many industrial applications requiring to be free of antibiotic resistance markers. Thus, the food-grade genome editing methods are needed for *B. subtilis*. Various counter-selectable markers such as *upp* (Fabret et al., 2002), *blaI* (Brans et al., 2004), *mazF* (Zhang et al., 2006), *araR* (Liu et al., 2008), and *hewI* (Wang et al., 2012) have been used to replace the antibiotic resistance markers in the genome editing of *B. subtilis*. However, the methods using *upp*, *blaI*, and *araR* require prior modifications on the second region of the chromosome and left traces of foreign DNA in the genome. The methods using toxic genes, such as *mazF* and *hewI* often generate undesired spontaneous resistant mutants. To overcome the limitation of the previous methods, a genome editing method using a synthetic gene circuit was developed (Jeong et al., 2015). Since CRISPR/Cas9-mediated genome editing methods have been widely spread, they have also been applied in *B. subtilis*, recently (Hong et al., 2018).

CRISPR/Cas9-mediated genome editing in *B. subtilis* has been carried out through chromosomal expression (Westbrook et al., 2016) or plasmid-based methods (Altenbuchner, 2016; Zhang et al., 2016; So et al., 2017). The chromosomal expression method enabled site-specific mutation, gene insertion, continuous genome editing, multiplexing and CRISPR interference (Westbrook et al., 2016). However, consecutive genome editing using the chromosomal integration system requires repeated restoration of the native *thrC* locus, which is the gRNA integration site. Furthermore, using toxic genes, such as *mazF* to restore the native *thrC* gene locus may result in undesirable spontaneous resistance mutations. An all-in-one system in which the *cas9*, gRNA, and donor DNA are assembled in a single plasmid was successfully used to delete the 25.1 and 4.1 kb DNA fragments from the genome and repair the *trpC2* mutation of *B. subtilis* 168 (Altenbuchner, 2016). Another all-in-one system was used to multigene disruption in undomesticated *B. subtilis* ATCC 6051a (Zhang et al., 2016). Although the single plasmid systems have been successfully used on the genome editing of *B. subtilis*, the large-sized plasmid may limit restriction enzyme sites for cloning and affect negatively to transformation and mutation efficiencies. We have also tried to use the all-in-one system. However, we obtained very poor mutation efficiency under our experimental condition. Thus, we modified the two-plasmid system previously reported for multi-round genome editing in *B. subtilis* (So et al., 2017).

All the plasmid-based approaches require the efficient replacement of the sgRNA-containing plasmid in multi gene editing. Plasmid curing has been commonly accomplished through serial subcultures in a non-selective medium and by screening for the loss of plasmid. The temperature sensitive origin could be introduced for an efficient exclusion of vectors; however, those methods are laborious and time-consuming. Otherwise, counter selection markers may be included in the plasmid backbone, but these tend to cause mutational escape and often require particular conditions for the medium and host (Reyrat et al., 1998). In this study, we introduced the sgRNA_st_ to remove the sgRNA-containing plasmid. To avoid the sgRNA-containing plasmid removal, prior genome editing, the sgRNA_ct_ should be generated before the sgRNA_st_. We accomplished the condition by using the *spoIVA* promoter, which is completely repressed in the growth phase, to synthesize the sgRNA_st_, while the sgRNA_ct_ was synthesized under the control of the constitutive promoter. Thus, the temporal separation of the two sgRNA syntheses during cultivation enabled the genomic editing and sgRNA-containing plasmid removal to take place in sequence, which automatically made the cells ready for the next round in multiple genome editing. Our efficient gRNA removal system can largely simplify the multi-round genome editing process in comparison with the traditional negative selection methods for plasmid curing. Since the *spoIVA* promoter is a σ^*E*^-dependent, our system is limited when the gene required for the activation of σ^*E*^ is the deleting target, such as *spo0A* and *sigF*. In that case, the stationary phase-specific promoter (Lee et al., 2010), the acetoin-regulated promoter (Silbersack et al., 2006) or phosphate starvation-inducible promoter (Qi et al., 1997; Choi and Saier, 2005) may be used for the synthesis of the sgRNA_st_ instead of the *spoIVA* promoter.

The curing efficiency using the plasmid containing both sgRNA_ct_ and sgRNA_st_ was reduced to 74%, while the plasmid including only one sgRNA_st_ showed 100% curing efficiency (Figure 3). We found that the curing efficiency using the plasmid containing both sgRNA_ct_ and sgRNA_st_ was restored to 98% when the expression of the *cas9* gene was induced by the IPTG. Thus, the reduction of the curing efficiency when using two sgRNAs may be due to insufficient amounts of Cas9 protein to produce sufficient sgRNA_st_/Cas9 complex at the sporulation phase, than when one sgRNA_st_ is used. A previous report showed that the leaky expression of the Cas9 without IPTG induction was enough to make an efficient mutation of the *B. subtilis* genome (So et al., 2017), suggesting that IPTG induction does not significantly change the mutation efficiency. The replication origin of the gRNA-containing plasmid was derived from the plasmid pTA1060 with a copy number of about 5 in *B. subtilis* (Bron et al., 1987). Our results suggest that the amount of Cas9 expressed by IPTG induction is sufficient to eliminate the 5-copy plasmid.

The self-curing system that we have developed here did not have the prerequisites, such as further gene sets and a host background. Although several inducible promoters have been reported for use in the *Bacillus* species (Bhavsar et al., 2001; Phan et al., 2006), they showed basal expression levels even without inducers. If the inducible promoters exhibiting leaky expressions are used, the sequential occurrence of genome editing, and plasmid self-curing may be difficult. The difficult tight-control of inducible promoters in the *Bacillus* species suggests that a sporulation-phase specific promoter is useful for the controlled expression of the sgRNA_st_. Although our system is proven through consecutive gene deletion, we believe that it is capable of performing all sorts of multiple genome editing, including point mutations and insertions. This system may be applied to other *Bacillus* strain engineering and would be helpful in academic research, industrial production, therapeutics, and agricultural applications. Also, other microorganisms other than *Bacillus* can easily perform multiple genome editing, if an appropriate stationary phase specific promoter is used for the sgRNA_st_ synthesis.

## Author Contributions

S-KC designed the experiments. HL and S-KC realized all experiments and wrote the manuscript. All authors reviewed the manuscript.

## Conflict of Interest Statement

The authors declare that the research was conducted in the absence of any commercial or financial relationships that could be construed as a potential conflict of interest.
